# Chromhidrosis Linked to Personal Care Products

**DOI:** 10.7759/cureus.66997

**Published:** 2024-08-16

**Authors:** Anthony J Marcelletti, Camryn Slone, Mindy N Baker, Chase L Wilson

**Affiliations:** 1 Dermatology, University of Kentucky College of Medicine, Lexington, USA; 2 Dermatology, Elkhorn Dermatology, Georgetown, USA

**Keywords:** personal care products, colored dyes, eccrine sweat glands, colored sweat, chromhidrosis

## Abstract

Eccrine chromhidrosis (CH) is a rare condition characterized by the excretion of colored sweat from eccrine glands. This case report contributes to the medical literature by highlighting two instances of eccrine CH linked to over-the-counter personal care products, an association not previously documented. These products contained FD&C Blue No. 1, D&C Red No. 33, and Ext. D&C Violet No. 2, which are known colorants in various consumer items. These cases underscore the potential for personal care products containing colored dyes to cause eccrine CH. The medical community and consumers must be vigilant about product ingredients to facilitate an accurate diagnosis and promote informed usage. Healthcare professionals should consider the role of colored personal care products in their differential diagnosis of CH to recognize and address potential risks effectively. These cases emphasize the need to actively include colored personal care products in medical considerations to ensure that healthcare practices and consumer awareness properly recognize and address potential risks associated with these products.

## Introduction

Chromhidrosis (CH) is a rare dermatologic condition marked by colored sweat; it was first reported by Yonge in 1709 [1]. It is primarily classified into eccrine, pseudoeccrine, and apocrine CH, with eccrine CH more commonly presenting as generalized colored sweating [2,3]. Eccrine CH appears histologically normal, typically caused by the exogenous coloring of clear sweat through the excretion of water-soluble agents like dyes and drugs [3,4]. Eccrine CH can manifest nearly anywhere on the body because eccrine glands, which produce dilute salty sweat, are widely distributed [4]. The prevalence of CH remains uncertain; among 50 documented case reports in the literature, none are linked to personal care products [5]. This report presents two cases of eccrine CH resulting from the use of over-the-counter personal care products.

## Case presentation

Case 1: Old Spice Body Wash

**Figure 1:**
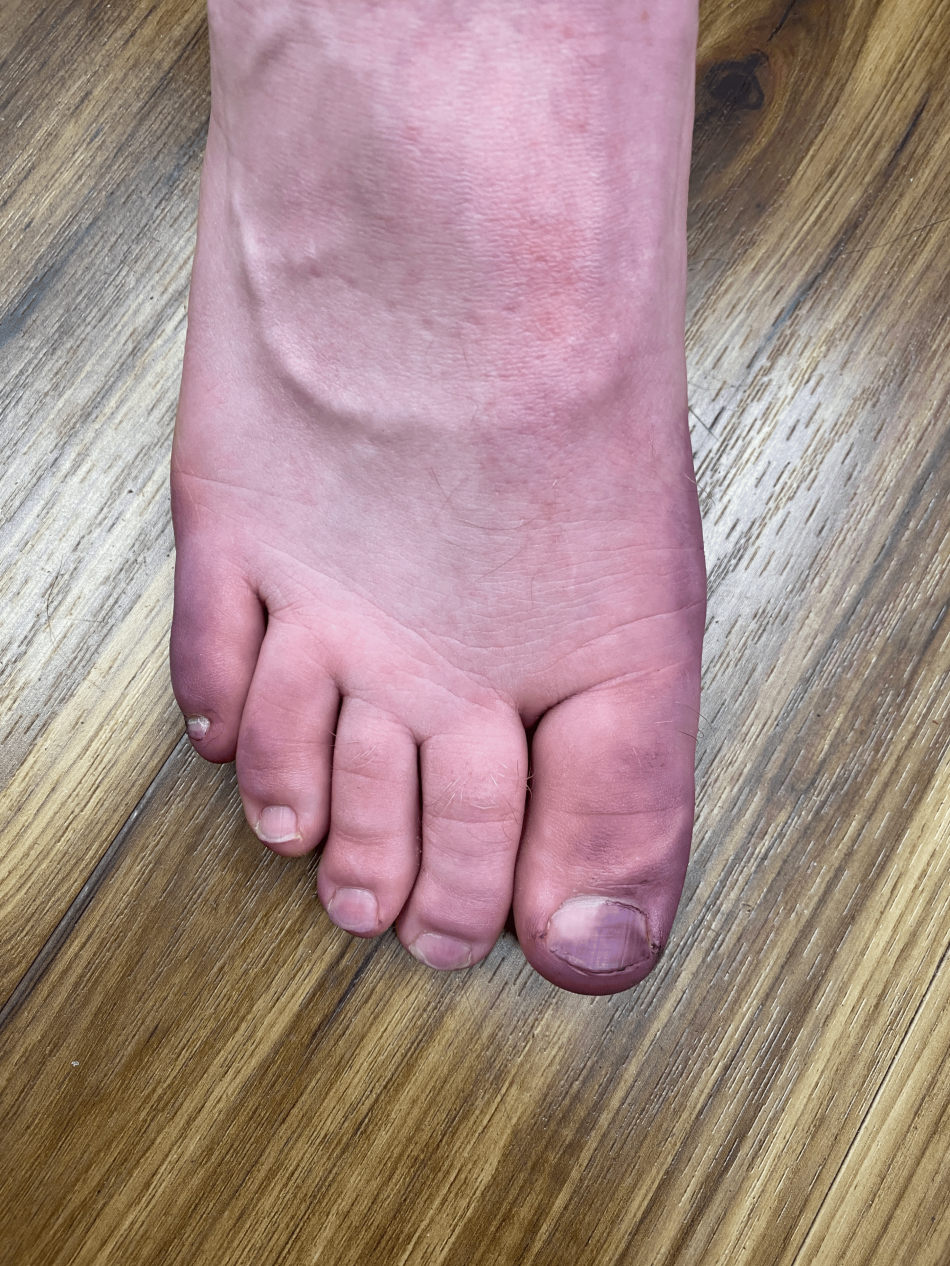
Case 1 - Blue discoloration was observed on the right distal plantar great toe

**Figure 2:**
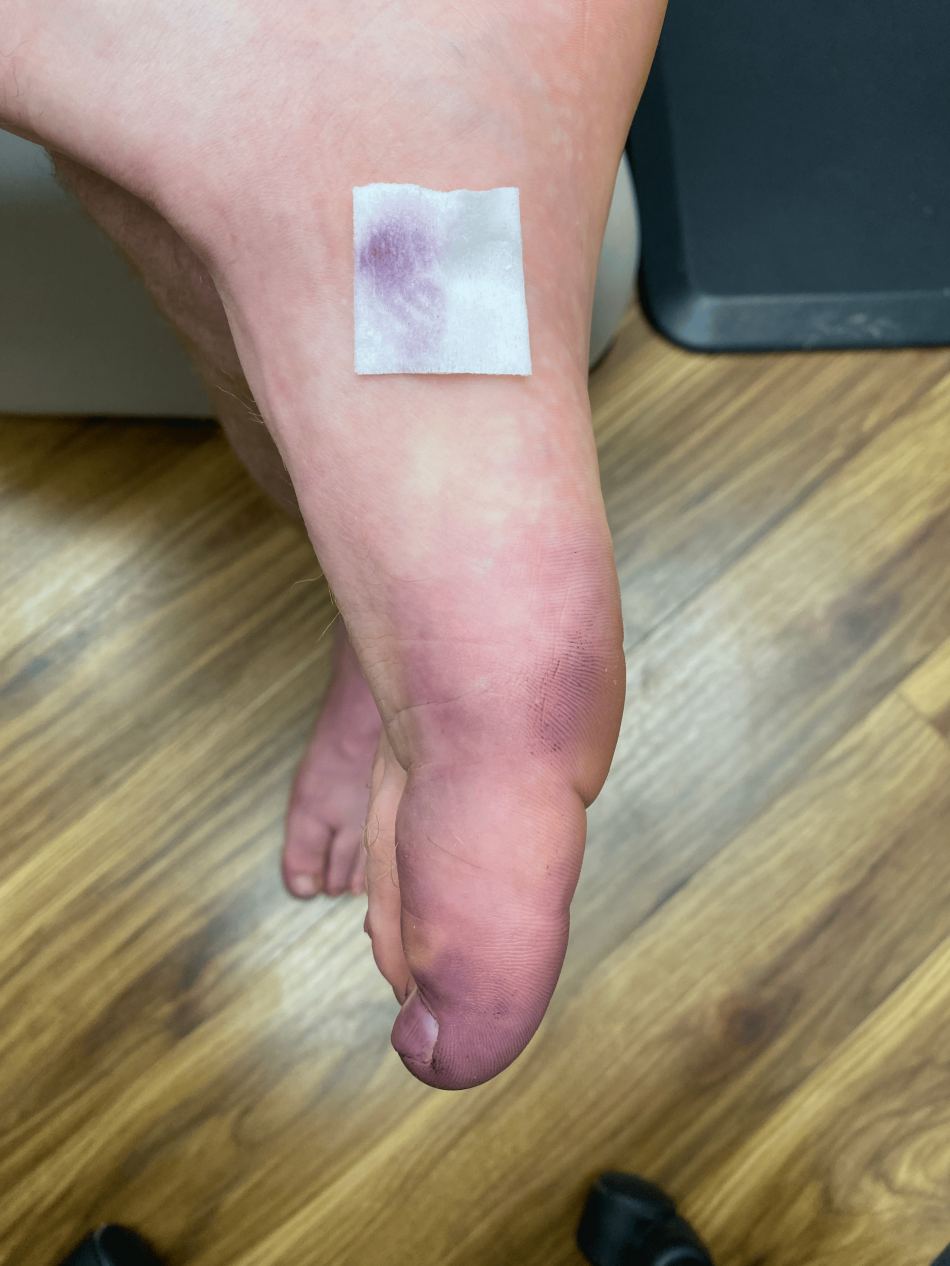
Case 1 - Rubbing affected areas on the right great toe with an isopropyl alcohol wipe removed the blue discoloration

A 26-year-old man presented to the clinic after he noticed blue discoloration in his socks after wearing his work boots. On examination, we observed blue-purple discoloration on the patient's left and right distal plantar great toes, which were easily wiped off with an alcohol swab (Figures 1-2).

**Figure 3:**
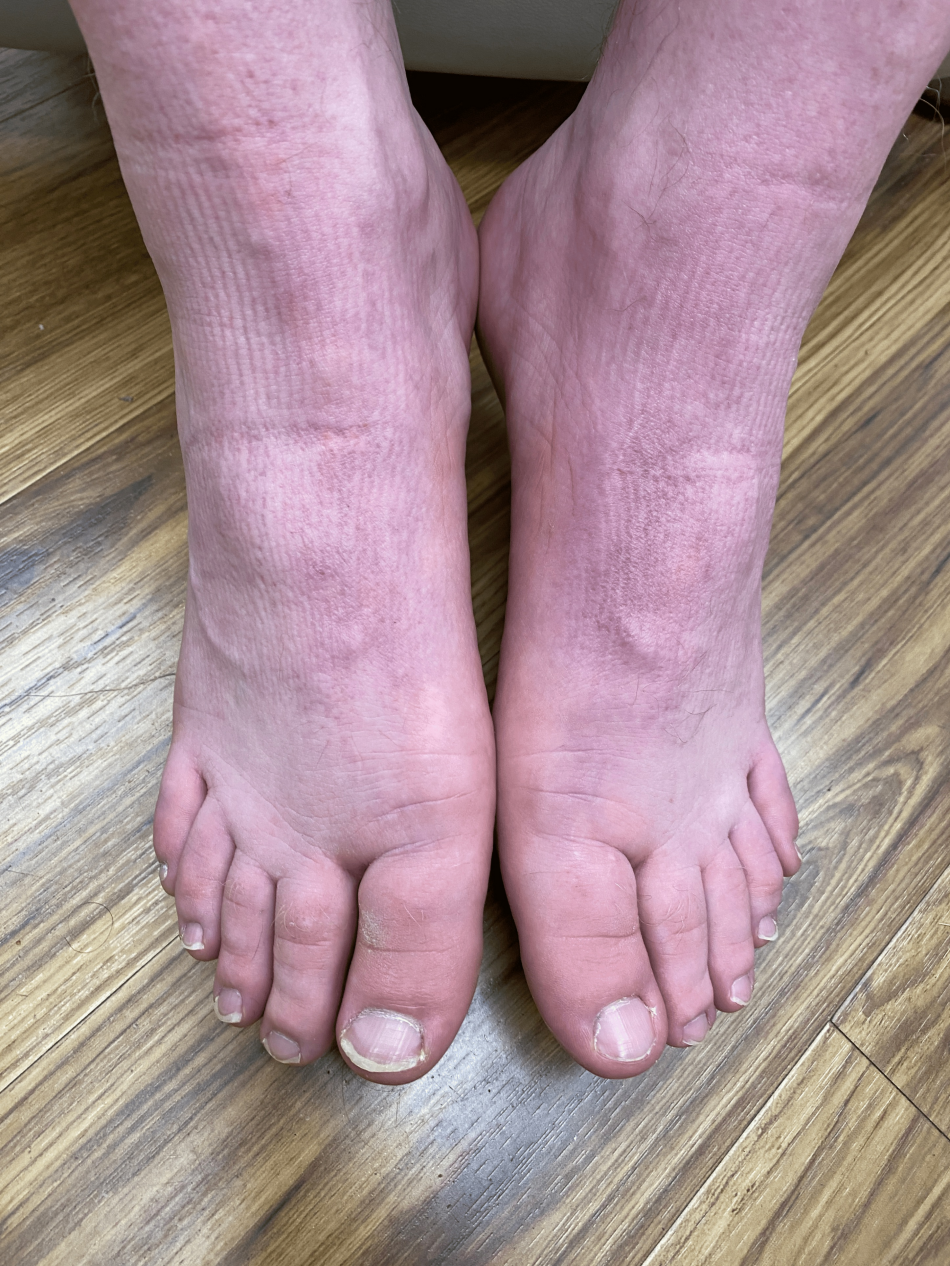
Case 1 - Absence of blue-purple discoloration at the patient's follow-up appointment

The patient reported using Old Spice Swagger with cedarwood body wash, which is blue-tinted, to wash his body. He did not specify how long he had been using the product before experiencing these symptoms. To rule out other potential causes, a thorough history and physical examination were conducted. There was no history of exposure to other dyes, medications, or chemicals that could cause the discoloration. There were no signs of infection, metabolic disorders, or systemic diseases. The patient was diagnosed with eccrine CH based on clinical features and instructed to discontinue the use of the suspected causative product. We advised him to return in six weeks for re-evaluation. The clinical exam at his follow-up appointment revealed the absence of any blue-purple discoloration, and the patient also noted that he no longer observed any discoloration (Figure 3).

This confirmed the cause-and-effect relationship between discontinuing the Old Spice Body Wash and the resolution of the chromhidrosis.

Case 2: Luseta Biotin & Collagen Purple Shampoo

**Figure 4:**
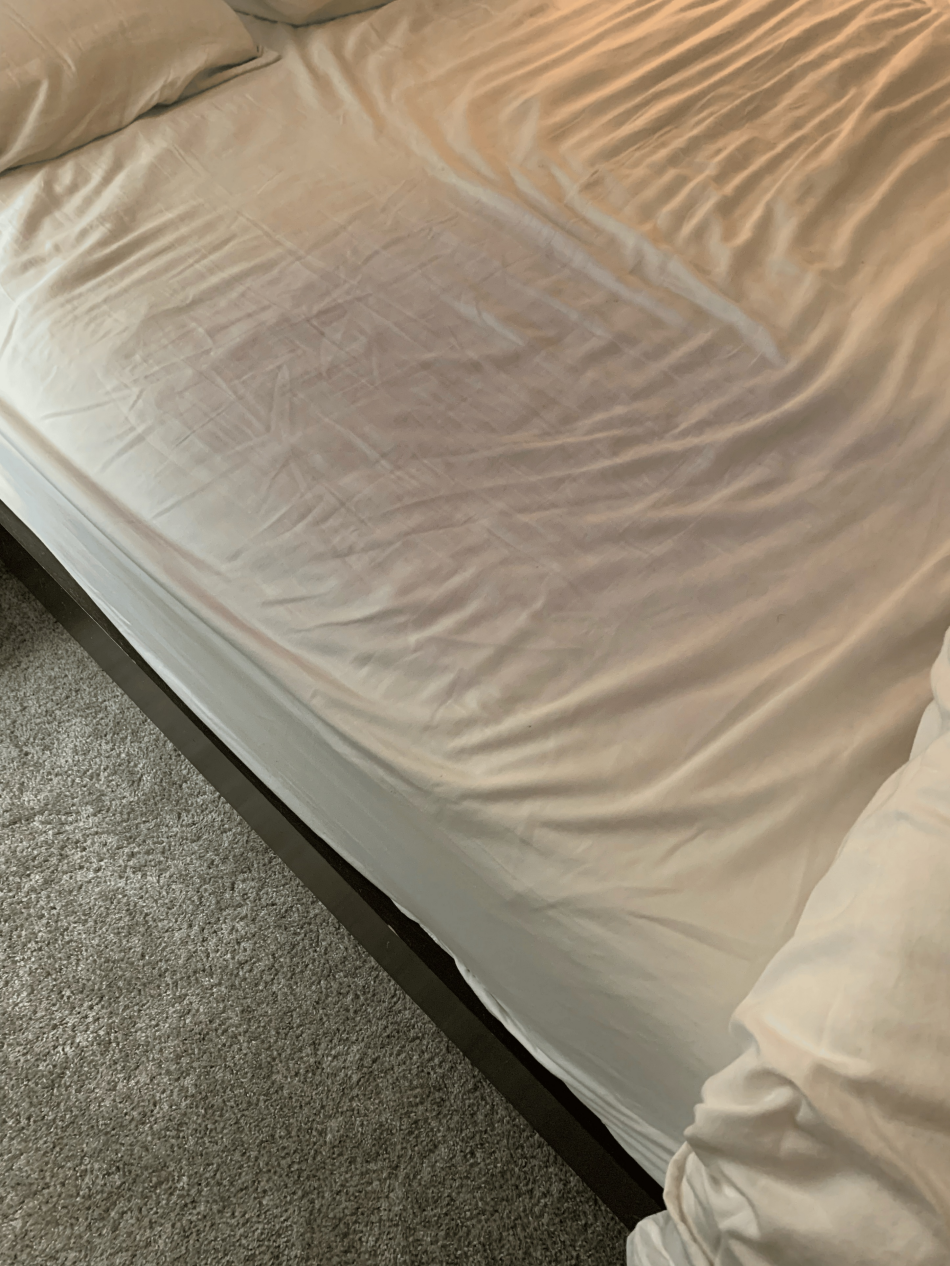
Case 2 - Contact with affected areas of the body led to blue discoloration transferring onto fabrics

A 31-year-old woman with no history of hyperhidrosis presented to the clinic after noticing a blue discoloration on her right posterior thigh, which appeared after sweating. She also noted that this discolored sweat was staining her clothing and furniture (Figure 4).

On examination, we observed blue discoloration in areas of sweat glands on the patient, distributed on the left proximal posterior thigh, right proximal medial posterior thigh, right proximal dorsal forearm, and left proximal dorsal forearm. All other relevant history and examination were unremarkable. The patient reported using a purple shampoo named "Luseta Biotin & Collagen Purple Shampoo/Conditioner for Hair Growth" on her hair two to three weeks before the blue sweat manifested. A comprehensive history and physical examination were performed to rule out other potential causes. The patient had no history of exposure to other dyes, medications, or chemicals. There were no indications of infection, metabolic disorders, or systemic diseases. Based on clinical features, she was diagnosed with eccrine CH, instructed to discontinue the use of the suspected causative product, and advised to return in six weeks for re-evaluation. The discoloration resolved after discontinuing the purple shampoo, confirming the diagnosis.

## Discussion

The presentation of eccrine CH may vary depending on the underlying cause. Krishnaram et al. reported one of the first cases of true eccrine CH presenting as yellow staining of clothes, which was concluded to be caused by tartrazine, a synthetic azo dye coating the bisacodyl the patient was taking [6]. Additionally, green sweat in patients diagnosed with eccrine CH has been observed as secondary to hyperbilirubinemia [7,8,9]. Red sweat has been attributed to coloring agents in tomato snacks and cranberry juice [3,9]. Our cases constitute the first known instances of CH following the use of widely available personal care products. 

We focus our examination on the ingredients in the products utilized by our patients. Old Spice Body Wash contains FD&C Blue No. 1, otherwise known as Blue 1, a synthetic organic compound used as a blue colorant [10]. Luseta Collagen & Biotin Purple Shampoo contains D&C Red No. 33 (Ci 17200), a synthetic dye produced from petroleum or coal tar sources, and Ext. D&C Violet No. 2 (Ci 60730), otherwise known as Acid Violet 43, a violet-colored coal tar dye [11]. FD&C Blue No. 1 exposure in the United States is significantly linked to its presence commonly found in juice, baking decorations/chips, and soft drinks [12]. D&C Red No. 33 is prevalent in cosmetics, particularly in toothpaste and mouthwash [13]. Acid Violet 43 is also frequently used as a cosmetic colorant in hair dyeing products [14].

Eccrine CH can be diagnosed through a thorough history and clinical evaluation [15]. Sweat color can be observed by wiping the skin with an alcohol wipe or examining stained white clothing, thus avoiding invasive procedures [15]. Following diagnosis, the assumed causative agent should be discontinued [15]. In both our cases, we diagnosed eccrine CH based on clinical features, and the diagnosis was confirmed when symptoms resolved after the patients discontinued the suspected causative products.

## Conclusions

CH, although benign, can be a diagnostic challenge and requires interdisciplinary efforts for effective outpatient treatment. A thorough history and physical exam should be performed on all patients presenting with colored sweat to aid in making the diagnosis, identifying the cause, and preventing unnecessary laboratory testing. Our cases align with existing knowledge that CH can manifest after the absorption of dyes, specifically FD&C Blue No. 1, D&C Red No. 33, and Ext. D&C Violet No. 2. These cases highlight that colored dyes, pigments, and chemical additives in personal care products may contribute to various health effects, such as CH. Hence, the medical community and consumers should have a thorough understanding of the ingredients in daily essentials. Vigilance about product ingredients not only aids in diagnosing similar presentations but also promotes informed product use. Also, there is a need to actively include colored personal care products in medical considerations, ensuring that healthcare practices and consumer awareness programs properly recognize and address potential risks associated with these products.
